# An In Vitro and In Silico Perspective Study of Seed Priming with Zinc on the Phytotoxicity and Accumulation Pattern of Arsenic in Rice Seedlings

**DOI:** 10.3390/antiox11081500

**Published:** 2022-07-30

**Authors:** Shuvasish Choudhury, Debojyoti Moulick, Muhammed Khairujjaman Mazumder, Binaya Kumar Pattnaik, Dibakar Ghosh, Lakshminarayana R. Vemireddy, Adil Aldhahrani, Mohamed Mohamed Soliman, Ahmed Gaber, Akbar Hossain

**Affiliations:** 1 Plant Stress Biology and Metabolomics Laboratory, Department of Life Science and Bioinformatics, Assam University, Silchar 788011, India; drubha31@gmail.com (D.M.); khairujjaman1987@gmail.com (M.K.M.); 2 Department of Zoology, Dhemaji College, Dhemaji 787057, India; 3 Symbiosis Institute of Geoinformatics, Symbiosis International (Deemed University), Pune 411016, India; binayapattnaik08@gmail.com; 4 Division of Agronomy, ICAR—Indian Institute of Water Management, Chandrashekarpur, Bhubaneshwar 751023, India; dghoshagro@gmail.com or; 5 Department of Molecular Biology and Biotechnology, Sri Venkateswara Agricultural College, Acharya NG Ranga Agricultural University, Tirupati 517502, India; vlnreddy@angrau.ac.in; 6 Clinical Laboratory Sciences Department, Turabah University College, Taif University, Taif 21995, Saudi Arabia; a.ahdhahrani@tu.edu.sa (A.A.); mmsoliman@tu.edu.sa (M.M.S.); 7 Department of Biology, College of Science, Taif University, Taif 21944, Saudi Arabia; a.gaber@tu.edu.sa; 8 Department of Agronomy, Bangladesh Wheat and Maize Research Institute, Dinajpur 5200, Bangladesh

**Keywords:** Zn x As, rice, molecular docking, oxidative stress, ROS

## Abstract

Arsenic (As) contamination of the rice agro-ecosystem is a major concern for rice farmers of South East Asia as it imposes a serious threat to human and animal life; thus, there is an unrelenting need to explore the ways by which arsenic stress mitigation could be achieved. In the present investigation, we explore the effect of zinc (Zn^2+^) supplementation using the seed priming technique for the mitigation of As-induced stress responses in developing rice seedlings. In addition to the physiological and biochemical attributes, we also studied the interactive effect of Zn^2+^ in regulating As-induced changes by targeting antioxidant enzymes using a computational approach. Our findings suggest that Zn^2+^ and As can effectively modulate redox homeostasis by limiting ROS production and thereby confer protection against oxidative stress. The results also show that As had a significant impact on seedling growth, which was restored by Zn^2+^ and also minimized the As uptake. A remarkable outcome of the present investigation is that the varietal difference was significant in determining the efficacy of the Zn^2+^ priming. Further, based on the findings of computational studies, we observed differences in the surface overlap of the antioxidant target enzymes of rice, indicating that the Zn^2+^ might have foiled the interaction of As with the enzymes. This is undoubtedly a fascinating approach that interprets the mode of action of the antioxidative enzymes under the metal/metalloid-tempted stress condition in rice by pointing at designated targets. The results of the current investigation are rationally significant and may be the pioneering beginning of an exciting and useful method of integrating physiological and biochemical analysis together with a computational modelling approach for evaluating the stress modulating effects of Zn^2+^ seed priming on As-induced responses in developing rice seedlings.

## 1. Introduction

Arsenic contamination of rice fields is an emerging concern for a wide spectrum of researchers working on the rice agroecosystem. The metalloid is considered a toxic element having no physiological role to play in the biological system. It is highly carcinogenic and its persistence in the environment has a severe hazardous impact on plants, animals and humans [1]. Arsenic (As) enters the environment from diverse sources such as arsenic mining and the use of arsenic-tainted underground water from irrigation [1]. Environmental contamination with arsenic has been reported across the continents of North America, Europe, East Asia and South East Asia, posing an extensive threat to agriculture and human health [2,3]. The rice agro-ecosystems of South East Asia are highly contaminated with As due to the extensive use of As-tainted ground water for irrigation [1,4,5].

Rice is a major cereal crop consumed by more than 50% of the global population and is considered the major dietary source of carbohydrates, essential elements and other nutrients. In South East Asia alone, rice and rice–related products account for more than 30% of the total daily dietary calorie requirement [6]. Arsenic accumulates in agricultural soils primarily due to the use of As-tainted water for irrigation [7,8]. Rice is predominantly cultivated under flooded conditions, which is a favorable environment for high As uptake, accumulation and mobilization that eventually results in a high grain As content [9,10,11,12]. The impact of arsenic phytotoxicity largely includes the inhibition of seed germination, the decline in plant growth and elongation, a loss of chlorophyll, nutrient imbalances, electrolyte leakage, raised levels of reactive oxygen species (ROS), oxidative stress, hormonal imbalances and the degradation of cellular metabolites [1,13,14,15,16,17]. Arsenate (As^5+^) is the most commonly found arsenic species in aerobic soils, while arsenite (As^3+^) is largely found in anaerobic conditions, particularly in flooded paddy fields [18,19,20]. Inside the cell, As^5+^ is reduced to As^3+^ in the cytoplasm [20,21], where it reacts with proteins, lipids and other biomolecules leading to an imbalance in cellular function and causing cell death [1,9,22]. The arsenate in the soil is reduced to arsenite as a consequence of microbial activity, which also aids its mobilization [23]. Inside the cell, arsenate is reduced to arsenite by the activity of the enzyme, arsenate reductase, which is widely observed in several plant species [24]. In comparison to As^5+^, As^3+^ is more toxic and reactive inside the cellular system, where it interacts with sulphydryl groups of proteins and enzymes leading to its functional loss [25,26]. Other inhibitory effects of arsenic include an inhibition of germination, loss of biomass, stunted growth, and photosynthesis inhibition that impairs plant growth and development [1,27,28,29]. Arsenic toxicity causes robust changes in plant metabolism by increasing the production of toxic chemical entities such as reactive oxygen species, which imparts oxidative stress by inactivating antioxidant defense machinery [29]. Consequently, As remains a major threat to plants and human nutrition; therefore, it remains very pertinent to develop strategies to mitigate the arsenic stress load in crop plants including rice.

In comparison to other cereals such as wheat, maize and barley, rice accumulates an exceedingly high (~10 folds) concentration of arsenic in its tissues and grain [21,30]. Studies on rice samples collected from different parts of the world revealed that the arsenic content in rice varies significantly in terms of speciation and concentration [31,32]. It has been noted that As is normally distributed in rice cultivated in developing economies, while the arsenic content is on the higher side in developed economies [31]. Over the decades, several mitigation strategies have been employed to mitigate abiotic stresses, including As stress in plants. Seed priming is the controlled hydration (soaking in water or a solution) of seeds for a predetermined period followed by drying and exposure to bright sunlight. This provides seeds’ internal metabolic activity a good start but prevents radicle emergence [33]. Conrath et al. [34] reported that as a result of the priming of seeds, the primed states are effective in facing many environmental odds and currently, seed priming is considered one of the most sought-after modes of improving abiotic stress tolerance, including the stress imposed by metal/metalloids, for its easy execution and cost-effectiveness as well as the fact that it can be executed in a wide range of plant species, including rice [35].

Micronutrients have a major role in stress mitigation and amelioration when applied in diverse ways and combinations. For example, iron (Fe) not only influences the dynamics and chemistry of arsenic in the rice agro-ecosystem but also mitigates stress responses by influencing various physiological and cellular responses [15,36,37,38]. Other elements, such as selenium and silicon, are also known to alleviate arsenic toxicity by influencing physiological and cellular responses in rice [12,39,40]. Zinc (Zn) is a chalcophile by nature and secures the 23^rd^ position in terms of richness in the earth’s crust [41]. Due to its unique nature of being a non-redox active element, Zn is efficient in executing a wide range of physiological functions by remaining as a key metallic ion for all of the six classes of enzymes and transcription factors [42,43,44,45,46,47]. In contrast, the role of Zn in As stress mitigation is seldom known and the impact of seed priming with zinc to mitigate As-induced stress responses in rice is not clearly understood.

In the stress biology of plants, focus has so far been given to delineating the effects of environmental toxins, including metals and metalloids, on morphology, yield, gene expression and antioxidant metabolism, and their amelioration. Consequently, these toxins might have the potential to affect vital enzymes, proteins and receptors directly by interfering with their activities, and it may be thought prudent that secondary metabolites and metal ions may ameliorate the same by excluding those toxins; however, studying such interactions in vitro and in vivo is a costly and time-consuming venture, and requires sophisticated instruments. This is where computational modeling finds tremendous use, which is already established as a powerful tool that may be used to predict the interactions of small molecules and ions (called ligands) with macromolecules such as enzymes and receptors. Molecular docking tools are employed to predict the direct interactions between such molecules. Using such computational tools, we have performed a few studies to predict the interactions of metal and metalloid ions on the vital enzymes of different crops [48,49,50,51], and the results were promising, which have opened a new avenue of plant stress research. In line with our earlier works, the present study uses molecular docking to predict the possible interactions of As^3+^ ions with four antioxidant enzymes of rice, viz. superoxide dismutase (SOD), catalase (CAT), glutathione peroxidase (GPx) and glutathione reductase (GR). Further, we have predicted whether Zn^2+^ and secondary metabolites, including AsA and GSH, may interfere with the interactions of As^3+^ and thereby ameliorate arsenic toxicity. 

Growth regulators and metabolites are essential components of plants that regulate diverse aspects of cellular functions and although the signaling roles of these entities during abiotic stress have been largely investigated, their specific functional roles during abiotic stresses are not entirely known. Further, the potential of metal ions in interacting with antioxidant enzymes and the role of plant secondary metabolites thereon, are poorly known. In the present computational modelling study, we investigate the role of Zn^2+^ in ameliorating As^3+^-induced stress, and the role of AsA and GSH as model antioxidant enzymes. The present investigation adopts physiological, biochemical and computational analyses to elucidate the basis of Zn^2+^ priming for the mitigation of As-induced stress responses in rice seedlings. The expected outcomes of the current investigation will serve as markers that will be highly useful for comprehending the role of Zn^2+^ priming in regulating As-induced phytotoxicity in rice. 

In the present study, we try to evaluate the efficacy of rice seedlings primed with Zn^2+^ when subjected to As stress on its possible role in quenching the redox imbalance and As-accumulation pattern. Furthermore, our efforts are also devoted to integrating the outcomes from “*wet lab*” analysis with the projections gathered from “*dry lab*” findings. The objectives of the current investigation are to understand (i) the redox homeostasis of rice varieties, (ii) the As-accumulation pattern and (iii) the prediction of the mode-of-action of Zn, when the seedlings of two rice varieties widely cultivated in the north-eastern part of India are grown in presence of As stress.

## 2. Materials and Methods

### 2.1. Plant Material and Treatments

For this investigation, two rice (*Oryza sativa* L.) varieties were considered. The seeds of the variety ‘*Kalajeera*’ were obtained from local farmers, in Silchar, India, while the seeds of the variety ‘*Gomti*’ were collected from A.N. Rangana University, Tirupati, India. The seeds were brought to the laboratory in sterile plastic bags and stored in a dry cool place. The seeds were surface sterilized with a 0.1% (*w*/*v*) mercuric chloride (HgCl_2_) solution followed by repeated rinsing in sterile distilled water (dH_2_O). The seed priming with zinc (Zn^2+^) was performed with two doses (0.5 mg L^−1^ and 1.0 mg L^−1^) of zinc chloride by soaking the sterile rice seeds for 24 h in the solutions. The control set contained only the surface-sterilized seeds. The Zn^2+^ seeds were air-dried before using [52]. Both the Zn^2+^ primed and non-primed rice seeds were germinated over moistened paper towels at 32 ± 2 °C for 48 and 72 h and the germinated seeds were hydroponically grown in plastic cups containing ~350 mL of Yoshida nutrient solution for a period of 2 d in a growth rack under white fluorescent light with a 16 h photoperiod at room temperature (32 ± 2 °C). The arsenic (As) stress was induced by treating the 2 days (d) old rice seedlings with 150.0 µM of sodium arsenate (Na_3_AsO_4_) supplemented with Yoshida nutrient solution and grown for a period of 5 d under the growth conditions as described above. After 7 d of growth under arsenic stress, root and shoot samples were harvested for analysis. Here in this investigation, each treatment combination (s) was subjected to a replicate five times (*n* = 5) in different cups. Each cup had eight healthy seedlings, arranged according to the complete randomized fashion.

### 2.2. Growth Responses and Total Chlorophyll Content

The growth responses in the rice seedlings were recorded in terms of their root and shoot elongation, fresh biomass and dry biomass. The root and shoot elongation were measured using a centimeter (cm) scale. The fresh biomass was measured as the fresh weight (FW) of the samples. For determination of the dry biomass, the fresh samples were oven-dried at 80 °C for 48 h and the dry weight (DW) was recorded. For determination of the total chlorophyll content in the rice seedlings, 0.5 g of fresh shoot samples were homogenized with 80% (*v*/*v*) cold acetone in a pre-chilled mortar pestle. The homogenate was centrifuged at 10,000× *g* for 10 min at 4 °C and the supernatant extract was collected for determination of the total chlorophyll content by measuring the absorbance at 663 and 645 nm.

### 2.3. Acid Digestion for Determination of Total As—Content

Fresh root and shoot samples were harvested and oven-dried at 80 °C for 24–48 h. The dried tissue samples were digested with 5 mL of an acidified mixture containing equal parts of 70% perchloric acid and nitric acid and then digested overnight at room temperature. The mixture was further digested over a hot plate at 80 °C until the acid solution turned completely clear. The final volume was adjusted to 20 mL with deionized water and the arsenic (As) content was determined using an atomic absorption spectrometer with an MHS system (AAnalyst 200, Perking Elmer, Waltham, MA, USA). During the course of the elemental profiling, we also analyzed the Rice Flour SRM-1568a to ensure the quality of the digestion and quantification process. Here we obtained ≥96% accuracy [39].

### 2.4. Determination of Hydrogen Peroxide, Superoxide Radical and Lipid Peroxidation

The ROS production in the rice roots and the shoots was measured in terms of hydrogen peroxide (H_2_O_2_) and the superoxide radical (O_2_^●^^−^) contents were examined by adopting the methodology of Sagisaka [53], Elstner and Heupel [54]. As an indicator of ROS-induced oxidative stress, the lipid peroxidation in the rice roots and shoots was measured as per the method of Heath and Packer [55] as the total malondialdehyde (MDA) content determined by a thiobarbituric acid (TBA) reaction.

### 2.5. Enzymatic Antioxidants

For the extraction of the antioxidant enzymes, 0.5 g of fresh tissue samples were grounded with liquid nitrogen and a homogenized ice-cold 0.1 M Na–phosphate buffer (pH 7.2). The homogenate was centrifuged at 16,000× *g* for 20 min at 0 °C and the supernatant extract was collected for determination of the activities of antioxidant enzymes such as catalase (CAT [EC 1.11.1.6]), guaiacol peroxidase (GPX [EC 1.11.1.7], superoxide dismutase (SOD [EC 1.15.1.1] and glutathione reductase (GR [EC 1.8.1.7]). The CAT activity was determined as per the method adopted by Chance and Maehly [56]. The SOD activity was determined as suggested by Beauchamp and Fridovich [57]. The GR activity was determined as per the method of Smith et al. [58].

### 2.6. Non-Enzymatic Antioxidants

The non-enzymatic antioxidants in the rice roots and shoots were measured in terms of the total ascorbate (AsA) and glutathione (GSH) content as per the methods suggested by Oser [59] and Griffith [60], respectively. All spectrophotometric measurements were made using a UV-Visible spectrophotometer (Lambda 35, Perkin Elmer, Waltham, MA, USA). 

### 2.7. Computational Modelling 

The receptors, namely, the crystal or NMR structures of SOD, CAT, GPx and GR were not available at the RCSB Protein Data Bank or NCBI MMDB databases; thus, the three-dimensional structures were obtained from the SWISS-MODEL database (https://swissmodel.expasy.org/ (accessed on 20 June 2022)), a database of protein structures predicted using homology modeling. The 3D structure of *Oryza sativa* Mn-SOD, bearing Accession no. Q43008, having 231 amino acids in a single chain, was downloaded in .*pdb* format. Likewise, the structure of the CAT of rice was downloaded from the database bearing Accession number Q10S82, having one chain with 492 amino acids. The homology modeled structure of GR was downloaded from the database bearing 492 amino acids in one chain, with Accession No. P48642. The 3D structure of GPx was downloaded from the database (Accession No. Q10L56), having 169 amino acid residues in a single chain. All the three-dimensional structures used in the present study have been predicted using the algorithm AlphaFold [61,62].

### 2.8. Molecular Docking

The structures of the receptors were loaded into the Molegro Virtual Docker (MVD) software individually, followed by the addition of the ligands to the workspace. Due to the lack of any reference or co-crystallized ligand, cavity detection was performed, using the ‘*van der Waals* surface’ as the parameter. Two cavities were detected for the Mn-SOD, of which the largest cavity was positioned at X: 11.7554, Y: 9.47629 and Z: −2.69671, bearing a surface area of 70.4 Å^2^ and a volume 15.36 Å^3^, was selected. The docking parameters included a MolDock scoring function with a grid resolution of 0.30 Å, and the binding site was selected around the detected cavity centered at X: 12.63, Y: 6.90 and Z: −3.75, and the amino acids within a radius of 15 Å were included. The docking was performed with 10 runs of 1500 iterations. Molecular surfaces for the docked ligands were developed with a resolution of 0.70 Å and probe radius of 1.00 Å, following standard protocols [50,51]. For the docking study using the target CAT, the largest cavity centered at X: 13.1807, Y: −12.3557 and Z: −17.0456, having a surface area of 2318.08 Å^2^ and a volume of 694.272 Å^3^, was selected. The docking was performed at the site using the same parameters as was used for SOD. For GPx, the cavity centered around X: 9.74504, Y: 17.712 and Z: −3.82037, having a surface area of 152.32 Å^2^ and a volume of 38.912 Å^3^, was selected for docking. For GR, the cavity centered around X: −6.98898, Y: 4.94197 and Z: −3.82902, with surface area of 1468.16 Å^2^ and volume of 526.336 Å^3^, was selected. The docking was performed at X: −7.75, Y: 5.75 and Z: −6.00 with the amino acids within a radius of 15 Å. The other docking parameters were the same as that of the Mn-SOD. The five best-docked poses of all the ligands, in terms of docking score, were considered for further analysis, following the methodology provided by Choudhury et al. [49].

### 2.9. Ligands

The structures of the ligands were obtained from the NCBI PubChem compounds database (https://pubchem.ncbi.nlm.nih.gov/ (accessed on 23 July 2022)). The ligands used for the docking against all the receptors were As^3+^ (CID 104734), Zn^2+^ (CID 32051), ascorbic acid (CID 54670067) and GSH (CID 124886). Against the Mn-SOD, its natural substrate, superoxide radical (CID 5359597), was used as a reference ligand. Against the CAT and GPx, the reference ligand used was hydrogen peroxide (H_2_O_2_) (CID 784).

### 2.10. Analysis of the Interactions of the Ligands

The docked poses of the ligands were loaded into the MVD workspace, and electrostatic surfaces were generated for each of the ligands for all the receptors. The surfaces were analyzed for overlapping among themselves. Furthermore, energy maps for the receptors were generated to observe the types of interactions (electrostatic, stearic and hydrogen bonds) that the surfaces of the receptors favored, and the region where the ligands had docked [51].

### 2.11. Statistics

The experiment was conducted in a completely randomized design with eight treatment combinations and replicated five (*n* = 5) times. To determine the treatment effect, ANOVA using the general linear model procedure was performed (*SAS* Windows Version 9.3). Treatment means were separated with the use of Tukey’s Honest Significant Difference test at a 5% level of significance.

## 3. Results

### 3.1. Zn^2+^ Supplementation through Seed Priming Effect on Rice Seedlings Grown under As Stress

The findings from our investigation suggest a noteworthy modulation in the morphological attributes, chlorophyll content, and As content visible in the rice seedlings. Upon exposure to As stress, an inhibition of root and shoot elongation was observed in the seedlings of both the tested rice varieties. The seedlings were grown in the presence of As stress and showed a 40 and 31% reduction in root elongation in ‘*Gomti*’ and ‘*Kalijeera*’, respectively, as compared to the controls. A similar reduction in the shoot elongation could also be seen in the seedlings of ‘*Gomti*’ (46.7%) and ‘*Kalijeera*’ (30.48%), respectively. Further, if we compare the responses exhibited by the two tested varieties for their relative susceptibility to As exposure in terms of root and shoot elongation, ‘*Gomti*’ seemed to be more prone to As stress with 1.66 and 1.87 times less root and shoot elongation compared to 1.45 and 1.43 times root and shoot elongation, respectively, shown by the ‘*Kalijeera*’ variety, on the date of observation. In terms of seedling elongation and varietal prospective, under As stress though, the root elongation was not of a significant type, but a significant (at a *p* ≤ =0.0007 level) impact of the varietal difference on shoot growth was visible (Table 1).

In terms of the fresh and dry biomass of the seedlings grown in the presence of As, they showed a declining trend as compared to the respective control(s) of both the rice varieties. The seedlings of ‘*Gomti*’ had 1.64 and 5.67 times less fresh and dry weight, respectively, in the roots, compared with the control. The seedlings of ‘*Gomti*’ also recorded 1.37 and 3.95 times less shoot weight than those of control, respectively. A similar reduction in the fresh and dry weight of the seedlings (roots and shoots) was also applicable to the ‘*Kalijeera*’ variety. From the statistical point of view, the fresh biomass accumulation pattern in the roots and shoots exhibited by the two tested varieties was highly significant (at a *p* ≤ 0.0001 level), indicating the striking inhibitory effects of As stress on developing rice seedlings (Table 1).

Among the doses of Zn considered here, supplementation with 1.0 mg L^−1^ resulted in 1.21 and 1.47 times and 1.44 and 1.07 times greater root length in the ‘*Gomti*’ and ‘*Kalijeera*’ varieties, respectively, compared to the root length of the seedlings grown in the absence of Zn under As-stressed conditions. The results also revealed that the supplementation with Zn resulted in the restoration of a greater shoot length by 1.10 and 1.31 times (1.0 mg Zn L^−1^) and 1.118 and 1.03 times (0.5 mg Zn L^−1^), when compared with the shoot length of the unprimed seedlings grown in the absence of Zn^2+^ in the ‘*Gomti*’ and ‘*Kalijeera*’ varieties, respectively (in the As-spiked growth medium). Supplementation with a 1.0 mg of Zn L^−1^ dose was more effective than 0.5 mg of Zn L^−1^, in restoring seedling growth.

After supplementing the seeds of both varieties using, the 0.5 mg Zn L^−1^ solution, the root of the seedlings recorded a 1.12 and 1.760 times greater fresh and dry weight than the roots of the unprimed seedlings grown in the presence of As stress, respectively. Likewise, 1.83 and 1.38 times more fresh and dry weights were also noted, respectively, in the shoots of those seedlings treated with 1.0 mg L^−1^ of Zn and grown in the presence of As stress in the ‘*Gomti*’ and ‘*Kalijeera*’ varieties, respectively, compared to the unprimed seedlings (Table 1). The adverse consequences of As exposure to young rice seedlings were reflected in a noteworthy reduction (significant at a *p* ≤ 0.0001 level) in the dry weight, observed in both the roots and shoots of the two tested varieties; however, in terms of the dry weight reduction in the roots though, the varietal difference was not of a statistically significant type, yet the decrease in the shoot biomass content displayed was significant for the varietal difference.

On the date of observation, the seedlings of ‘*Gomti*’ (primed with 0.5 Zn mg L^−1^) had a 1.09 and 1.32 times more fresh and dry weight, respectively, than the shoots of the unprimed seedlings grown in the presence of As stress. In the shoots of the seedlings of the ‘*Kalijeera*’ variety (primed with 0.5 Zn mg L^−1^), a 1.63 and 1.58 fold greater fresh and dry weight was observed, respectively, over the shoots of the unprimed seedlings grown in the presence of stress alone. A similar restorative trend in the fresh and dry weight patterns was also observed in the seedlings primed with 1.0 mg L^−1^ grown under As stress as compared to the unprimed seedlings (Table 1).

In the case of the total chlorophyll content, both the rice varieties experienced a drastic reduction upon exposure to As stress. On the day of observation, a 43.73 and 48.35% decline in the chlorophyll content was recorded in the second leaves of ‘*Kalijeera*’ and ‘*Gomti*’, respectively, grown in the presence of As stress as compared to the controls. The reduction in total chlorophyll content of both the tested varieties upon exposure to As stress was non-significant; however, the leaves of the rice seedlings primed with Zn exhibited an upward trend over those unprimed seedlings grown in the presence of As stress. Among the two varieties considered here, ‘*Gomti*’ (1.72 times) had an edge over the ‘*Kalijeera*’ variety (1.10 times)—both primed with 1.0 mg Zn L^−1^)—in terms of the restoration in chlorophyll content, compared to the unprimed seedlings also grown in the presence of As stress (Table 1).

### 3.2. Zn^2+^ Supplementation through the Seed Priming Effect on As-Accumulation in Rice Seedlings

When compared with the As-accumulation pattern in the Zn unprimed rice seedlings, two different trends were observed. In the case of the variety ‘*Gomti*’, seeds primed with 0.5 and 1.0 mg of Zn L^−1^ had a 1.17 and 1.47 times less As content, respectively, as compared to the As content in the root of unprimed seedlings grown in the As-stressed conditions. In the ‘*Kalijeera*’ variety, there was a 1.07- and 1.03-fold less As content exhibited by 0.5 and 1.0 mg of Zn L^−1^, respectively, compared to the As content in the roots of the unprimed seedlings grown in similar As-stressed conditions. The ‘*Gomti*’ variety, first showed an increase in As-content by 1.24 fold, followed by a recorded decrease by 1.03 times in the shoot As-content, when compared to the shoot As-content of the unprimed seedlings grown in As-stress conditions (Table 1). Apart from these, a significant (at a *p* < 0.0001 level) varietal impact on the As content in the growing seedlings was clearly visible. 

### 3.3. Zn^2+^ Supplementation through the Seed Priming Effect on As-Induced Oxidative Stress in Rice Seedlings

The rice seedlings were exposed to As stress and showed a sharp rise in stress-induced biomarkers in both the roots and shoots as compared to the controls. Among the biomarkers considered were, ROS (H_2_O_2_ and O_2_^●^^−^) and lipid peroxidation (as the MDA content), all showing a significant rise in levels during the As stress. Whereas the stress-responsive enzymatic responses exhibited a marked reduction (Table 2). Upon the exposure to As stress, 5.45–7.23 and 6.22–8.38 fold increases in the H_2_O_2_ content in the roots and shoots of the ‘*Gomti*’ and *Kalajeera* varieties were observed compared to the controls, respectively. About a 9.53–4.26 fold increase (roots) and 8.97–5.51 fold increase (shoots) in O_2_^●^^−^ could be seen in the ‘*Gomti*’ and *Kalajeera* rice varieties as compared with the controls, respectively. Compared to the MDA content (used to assess the lipid peroxidation levels) in both the roots and the shoots of the controls, the two tested varieties (‘*Gomti*’ and *Kalajeera*) exposed to As stress had a significantly greater MDA content in the range of 5.56–6.96 and 4.84–6.86 times greater, in their roots and shoots, respectively (Table 2). Regarding the As-induced ROS generation upon As exposure, the varietal difference was prominent. Among the two tested varieties considered here, the ‘*Kalajeera*’ variety had a comparatively higher amount of H_2_O_2_ and MDA content than ‘Gomti’ on the date of observation. 

The seedlings of the Zn-primed seeds (grown in As stress) exhibited quenching effects associated with minimizing the H_2_O_2_, superoxide radical and MDA content in both the roots as well as in the shoots, compared to the unprimed seedlings under similar stress conditions. This reduction in the H_2_O_2_, O_2_^●^^−^ and MDA content was valid for both tested varieties. The seedlings primed with 0.5 mg L^−1^ of the ‘*Gomti*’ variety after growing in the As-spiked solution had a 2.18–1.57 and 1.46–1.57 fold less H_2_O_2_ and O_2_^●^^−^ content in their roots and shoots, respectively, compared to the unprimed seedlings grown in a similar As-spiked solution. The seedlings of the ‘*Gomti*’ variety also had a 2.28- and 1.66 times less MDA content in their roots and shoots, respectively, compared to the unprimed seedlings. Apart from these, on the date of observation, the ‘*Gomti*’ variety had 1.77–1.83, 2.66–4.49, 2.29–3.03 and 5.13–6.43 times less CAT, GPX, SOD and GR activity, respectively, observed when compared with the controls. A similar trend (reduced) in the enzymatic activity can also be seen in the *Kalajeera* variety (Table 2).

### 3.4. Zn^2+^ Supplementation through the Seed Priming Effect on Antioxidants Metabolism in Rice Seedlings 

Apart from stress-induced biomarkers, the profiles of antioxidative enzymes were also considered here. The findings suggest that upon exposure to As stress, a drastic reduction in the enzymatic activity was observed when compared with the controls, in both the roots as well as the shoots of the seedlings of both the tested varieties. When compared to the controls, in the roots and shoots of the seedlings exposed to As stress, there was recorded a 61.32 to 59.25% and 64.25 to58.8% less AsA levels in the ‘*Gomti*’ and ‘*Kalijeera*’ varieties, respectively. The seedlings of the ‘*Gomti*’ variety exposed to As stress had 70.9–75.73% less GSH content in the roots and shoots, respectively, than the controls. A similar reduction in the GSH content in the roots and shoots (49.08–80.76%) of the seedlings of the ‘*Kalijeera*’ variety was observed when compared with the controls (Table 3). In terms of the varietal prospective under As-stressed conditions in the unprimed seedlings, the ‘*Gomti*’ variety exhibited significantly less AsAa activity (at a *p* < 0.05 level), over the ‘*Kalijeera*’ variety when compared with the controls. A similar trend of significantly less GSH activity upon As exposure also applied to the seedlings (roots and shoots taken together) of the ‘Gomti’ < Kalijeera’ varieties when compared with the controls.

When the AsA content of the Zn-primed seedlings (primed with 0.5 and 1.0 mg L^−1^) was compared with the unprimed seedlings that experienced similar As stress, there was a significantly higher content by 14.01–134.95% (roots) and 55.19–87.97% (shoots), respectively. A similar enhancement in the AsA activity was also observed in the ‘*Kalijeera*’ variety, with a 53.33–87.27% and 36.75–98.42% greater activity exhibited by the seedlings primed with 0.5 and 1.0 mg of Zn L^−1^, respectively, compared with the unprimed seedlings exposed to As stress (Table 3). 

An identical trend of the restoration of the antioxidant metabolism in both the tested varieties, primed with Zn solutions and grown in As-stressed conditions, was observed when compared with the unprimed seedlings. The seedlings of the ‘*Gomti*’ variety primed with 0.5 and 1.0 mg of Zn L^−1^ recorded 2.49–3.28 times and 1.42–2.72 times greater GSH levels in the roots and shoots, respectively, compared to the GSH levels recorded in the unprimed seedlings. About a 1.19–2.46 fold (in the roots) followed a by 2.39–2.45 fold (in the shoots) higher GsH content was exhibited by the seedlings of the ‘*Kalijeera*’ variety primed with 0.5 and 1.0 mg of Zn L^−1^, respectively, than the unprimed seedlings exposed to As stress (Table 3).

### 3.5. Zn^2+^ Supplementation through the Seed Priming Effect on Docking Scores and Their Interactions

The natural substrate of Mn-SOD, a superoxide radical, showed the lowest docking score among the five ligands tested. The As^3+^ and Zn^2+^ showed almost the same docking score, while the AsA and GSH had quite higher docking scores, highest in the case of the GSH (−114.497 kcal/mol). Except for the As^3+^ and Zn^2+^, the other ligands showed hydrogen bonding scores, which is an indication of the accuracy of the docking study, since the ions lacked any hydrogen bond donor or acceptor group(s). The docking scores (MolDock) of the AsA and GSH were found to be 4.07 and 5.78 - folds higher than that of the As^3+^ (Table 4). 

**Figure 1:**
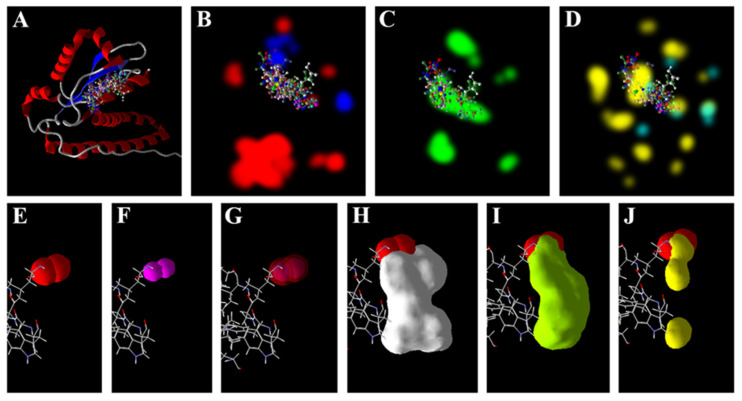
Docking poses and interactions of Mn-SOD with different ligands. (**A**) Docked poses of all ligands at the cavity; surfaces of the receptor favoring different interactions: electrostatic (**B**); stearic (**C**) and hydrogen bond (**D**). The electrostatic surfaces of As^3+^ (**E**); Zn^2+^ (**F**); and overlapped surfaces of As^3+^ and Zn^2+^ (**G**), As^3+^ and five poses of GSH (**H**); As^3+^ and ascorbic acid (**I**), and As^3+^ and superoxide radicals (**J**). Poses and surfaces were developed using MVD.

The docking of Mn-SOD returned two poses for both the As^3+^ (Figure 1E) and Zn^2+^ (Figure 1F), which were found to bind at two nearby regions of the receptor. Interestingly, it was found that the electrostatic surfaces of the As^3+^ and Zn^2+^ overlapped with one another (Figure 1G), although the surface of the former was larger. This indicates that both the ions may interact with the enzyme and that Zn^2+^ may interfere with the interactions of As^3+^. Moreover, the other ligands, AsA and GSH, may also have interfered with the interactions of the As^3+^ with the Mn-SOD, as they were predicted to have overlapping surfaces (Figure 1). The results of the docking study with the CAT revealed that H_2_O_2_ showed the lowest docking (MolDock) score while the GSH showed the best score. The Zn^2+^ and As^3+^ showed lower docking scores than the metabolites, but more than that of the H_2_O_2_. Both the ions showed no hydrogen bond scores, being devoid of any hydrogen bond donor or acceptor group(s). The docking scores of the Zn^2+^, AsA and GSH were found to be 0.95, 3.00 and 3.92 - folds higher than that of the As^3+^, respectively (Table 4). 

**Figure 2:**
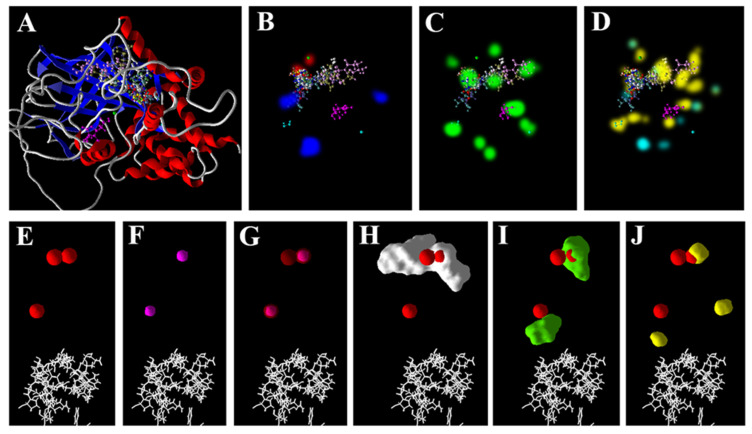
Poses and interactions of ligands and catalase: (**A**) Docked poses of all the ligands. The surface of the receptor favors (**B**) electrostatic, (**C**) stearic and (**D**) hydrogen bond interactions. Electrostatic surface of (**E**) As^3+^, (**F**) Zn^2+^, and overlapping surfaces of (**G**) As^3+^ and Zn^2+^, (**H**) As^3+^ and GSH, (**I**) As^3+^ and AsA, and (**J**) As^3+^ and H_2_O_2_. Poses and surfaces were developed using MVD.

On generating the electrostatic surfaces for the docked ligands at the active site of the CAT, all the ligands were found to bind in and around the cavity (Figure 2A). The As^3+^ was found to bind at three locations inside the pocket (Figure 2E), and the Zn^2+^ was found to bind at two (Figure 2F), while both the sites of the binding of the Zn^2+^ overlapped with two of the binding sites of the As^3+^ (Figure 2G). Likewise, the binding sites of the GSH (Figure 2H) and AsA (Figure 2I) overlapped with one of the three sites of the binding of the As^3+^. One of the sites of the binding of As^3+^ overlapped with that of the substrate, hydrogen peroxide (Figure 2J), indicating that the ion may interfere with substrate binding.

In the docking using GR, the GSH showed the highest docking score, and the As^3+^ showed the least docking score, which was, however, comparable to that of the Zn^2+^. Both the ions showed no hydrogen bond score. The docking score (free energy of the bonding) of Zn^2+^, AsA and GSH were found to be 1.02, 2.88 and 4.19 - folds higher than that of the As^3+^ (Table 4). Oxyglutathione was also used as a ligand against the GR, which, however, did not dock. 

**Figure 3:**
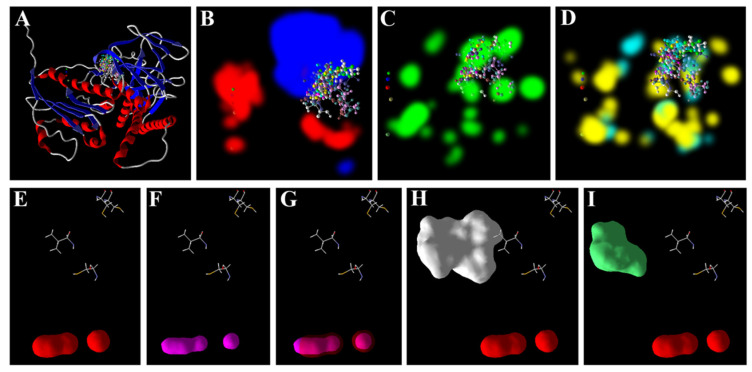
Docking poses and interactions of GR of rice with ligands: (**A**) poses of all ligands; regions of the receptor along with docked ligands favoring (**B**) electrostatic, (**C**) stearic and (**D**) hydrogen bond interactions. van der Waals surfaces of (**E**) As^3+^, (**F**) Zn^2+^, and overlapped surfaces of (**G**) As^3+^ and Zn^2+^. It may be noted that the surfaces of AsA (**H**) and GSH (**I**) do not show any overlapping with that of As^3+^. Poses and surfaces were developed using MVD.

The analysis of the docked poses of the ligands at the active site of the GR revealed that the ions docked at a different site than that of the metabolites (AsA and GSH) (Figure 3A). The ions were predicted to prefer surfaces of the receptor favoring electrostatic interactions, while the metabolites prefer regions favoring stearic and hydrogen bonding interactions (Figure 3B–D). Nevertheless, the docking sites of As^3+^ and Zn^2+^ overlapped with each other completely (Figure 3E–G), while as expected there was no such overlapping of the surfaces with AsA or GSH (Figure 3H–I).

**Figure 4:**
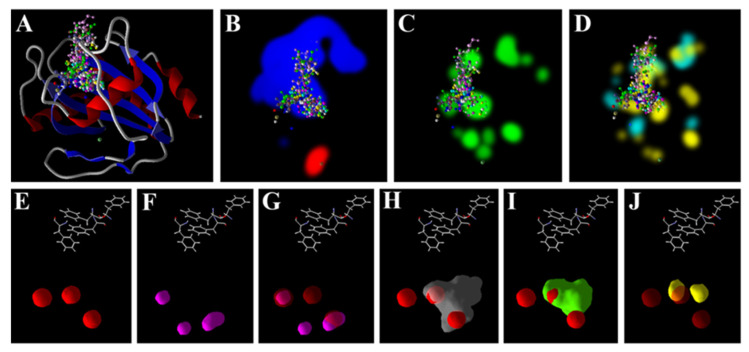
Docking poses and interactions of GPx of rice. (**A**) All docked ligands at the active site; surfaces of the receptor (along with the docked ligands) that favor (**B**) electrostatic, (**C**) stearic and (**D**) hydrogen bond interactions. Electrostatic surfaces of (**E**) As^3+^, (**F**) Zn^2+^, and overlapped surfaces of (**G**) As^3+^ and Zn^2+^, (**H**) As^3+^ and GSH, (**I**) As^3+^ and AsA, and (**J**) As^3+^ and H_2_O_2_. Poses and surfaces were developed using MVD.

In the case of GPx, the GSH showed the highest docking score, while the H_2_O_2_ showed the least score. The scores of the As^3+^ and Zn^2+^ were predicted to be the same, while the scores of the AsA and GSH were 3.26 and 4.62 - folds higher than that of the As^3+^ (Table 3 and Table 4). At the active site of the GPx, the Zn^2+^ and As^3+^ were found to bind at the surface of the receptor that favored electrostatic interactions, while the metabolites preferred to bind at the regions favoring stearic interactions. Both the As^3+^ (Figure 4E) and Zn^2+^ (Figure 4F) were found to bind at the same site of the enzyme; however, the other ligands were bound at a different location. On developing *van der Waals* surfaces for all the ligands, it was found that the surfaces of the Zn^2+^ and As^3+^ overlapped completely with each other (Figure 4G); however, there was no such overlapping in the case of the GSH (Figure 4H) and AsA (Figure 4I) with the As^3+^.

## 4. Discussion

Arsenic contamination of the rice agro-ecosystem is a major challenge for researchers from various domains working on it. In comparison to other cereals, rice is highly efficient in accumulating significantly higher levels of arsenic in its aerial parts and grains, thus altering the rice grain quality and mineralogical profile [12,63]. This poses a direct threat of arsenic poisoning in humans and animals due to the consumption of arsenic-tainted rice grains and animal fodder [1,52]. The north-east states of India are also reported to be contaminated with elevated As content. The significant presence of elemental contamination including As in the ground waters of the Brahmaputra floodplain, spanning across Assam (Lakhimpur and Diphu), Manipur (Kakching, Imphal East, Imphal West, and Bishnupur), Meghalaya, Mizoram, Nagaland, and Tripura further extended to Arunachal Pradesh, has also been confirmed by several earlier findings [64,65,66,67,68,69,70,71,72]. Thus, it will be necessary to assess the phytotoxicity of As on the popular rice varieties among the farmers of north-east India and this will be helpful in proposing a suitable mitigation (to minimize the As toxicity) plan for the region.

Globally, >30% of soils are underprovided for in terms of phyto-available Zn [73]. Cereals are regarded as susceptible to being Zn deficit, which often leads to a significant reduction in the yield and nutritional quality [74]. Fageria et al. [75] and Quijano-Guerta et al. [76] found that the shortfall of Zn often considered a major stress factor in the irrigated rice agro-ecosystem, is even greater than the global average and may be extended by up to 50%. The redox potential, along with the presence of sulfur are considered important for the relatively low phyto-availability of Zn. The precipitation of Zn enhances with the depressing redox potential, which later trims down the phyto-availability of Zn in the rice agro-ecosystem [77,78]. 

### 4.1. Effect of Zn^2+^ Supplementation on As-Induced Morpho-Physiological Responses and As Accumulation

Seed priming is one of the foremost methods for mitigating the abiotic stress load in plants [79]. For rice grown under stressful conditions, it is considered as a useful mitigation strategy even at the early seedling stage also [80]. Our findings indicated that rice seed priming with Zn^2+^ has a substantial impact on rice seedlings during As stress. Besides alleviating the toxic impact of As on the growth and biomass, it also restricts a high As accumulation, with a marked impact on ‘*Gomti*’ as compared to the rice variety ‘*Kalijeera*’. In terms of evaluating the impact of Zn^2+^ supplementation on the growth and biomass, the responses of both the rice varieties were relatively alike. While the chlorophyll content was significantly reduced under As stress in the absence of Zn^2+^ priming, the primed seeds of ‘*Gomti*’ had a higher chlorophyll content as compared to ‘*Kalijeera*’ during As stress (significant at a *p* < *0.05* level). Germination and early seedling development are usually more prone to metallic toxicity, and early seedling development is a crucial step in toxicity assessments [81]. Upon exposure to As stress, a greater oxidative stress was seen in terms of a significantly (moderate to high level) greater H_2_O_2_, O_2_^●−^ and MDA content in both the roots and shoots compared to the controls, which supports the observations laid by previous observations [82]. The consequences of greater oxidative stress resulting in the decrease in the chlorophyll content indicate lower photosynthesis as the oxygen turned into an electron acceptor, and further, the metabolite ROS [14] (Table 1 and Table 2). This reduction in chlorophyll content is in good agreement with the finding of Moulick et al. [40], who stated that it might be due to a hindrance in chlorophyll biosynthesis by modulating chlorophyll synthase [83]. Observations made by Rahman et al. [84], who indicated a positive correlation between the chlorophyll content and shoot growth, supports the findings from the present investigation with a reduced shoot length in the unprimed seedlings. The reduced shoot growth might have been a consequence of lesser carbohydrate generation and/or allocation resulting in a decrease in the shoot development (Table 1, Table 2 and Table 3). 

In the current investigation, exposure to As caused an enhancement in the As content in the roots and shoots of the unprimed seedlings, which was lessened by two different doses of Zn, used for the seed priming (Table 1). Conversely, the reduction in the As content was not of a significant type, i.e., variety x seed priming, yet the drop in the As content among the Zn^2+^-primed seedlings might be credited to the electrostatic interactions among the As and Zn, which in turn enabled a comparatively less soluble compound (As-Zn) formation; therefore, lessening the phyto-availability of As to the rice seedlings. Another important fact emerges from the current investigation in that the varieties responded differently to Zn^2+^ to minimize the As content in the seedlings, compared to the unprimed seedlings. The reduction in As content in the rice plants by the application of Zn is in good agreement with previous observations [17,85,86] (Table 1).

### 4.2. Effect of Zn^2+^ Supplementation on As-Induced ROS Accumulation, Oxidative Stress and Antioxidant Metabolism in Rice Seedlings

Greater ROS production causes significant damage to the plant, altering its ability to protect against such ROS-induced oxidative stress [87]. The SOD is considered a first-line defense enzyme that helps the conversion of more toxic O_2_^●−^ into the comparatively less toxic H_2_O_2_ [88]. The results support the idea behind the lesser activity of SOD in both the tested varieties, which can be attributed to the consequences of exposure to a greater As content. In another scenario comparing the SOD content of Zn^2+^-primed seedlings with unprimed seedlings, an increase in SOD activity could be seen in both the tested varieties. The greater SOD activity in the Zn^2+^ primed seedlings indicated a stimulating effect of the SOD activity, even under As-stressed conditions. The responses of both the varieties of rice grown under As stress clearly revealed its susceptibility to metalloids; however, the Zn^2+^ primed rice seedlings responded by rejuvenating their morpho-physiological traits along with a stimulation of their biochemical defense mechanisms.

It is known that several plant secondary metabolites, including AsA and GSH, confer protection against metal/metalloid-induced abiotic stresses [89,90,91,92]. Further, an increase in the levels of several metabolites including AsA and GSH have been widely reported to ameliorate abiotic stresses in plants. One of the major mechanisms underlying the amelioration of such stress is the maintenance of the redox status. The AsA-GSH system is the major antioxidant defense mechanism in plants, and the redox status in this antioxidant system is maintained by enzymes. It is, therefore, thought prudent to investigate whether these metal and metalloid ions may directly interact with these enzymes and if the metabolites may prevent and/or interfere with the interaction of the ions. Here, the impact of seed priming with Zn^2+^ bears a significant influence in modulating the AsA-GSH system under As stress, which might be considered as a mode of action behind the Zn x As.

### 4.3. Molecular Docking Analysis of Zn × As Interaction by Targeting Antioxidant Enzymes 

Molecular docking is a very useful computational modelling tool to predict the interactions of small molecules with an enzyme, channel protein or receptor, and is extensively used in drug discovery/repurposing research. When a ligand binds to an enzyme’s active site, it may prevent the binding of another, which depends on the affinities, which are measured as docking scores. The docking scores represent the amount of energy liberated when a compound docks/binds with an enzyme. A ligand having a higher docking score (i.e., a more negative score), may potentially prevent another which has a lower (i.e., a less negative) docking score to interact with a receptor. Compared to other similar modelling algorithms, the MVD has better accuracy [93].

Of late, we have used molecular docking tools to predict the possible interactions of metal/metalloid ions with such targets of different plants [48,49,50,51]. In the present study, in vitro analysis revealed that As^3+^ may affect the antioxidant system of rice, and thus, it was thought prudent to perform an in silico analysis. MVD, used in the present study, is a very useful molecular docking software that predicts the interactions of ligands and receptors with over an 87% accuracy [93]. The present results indicate that As^3+^ may potentially interact with the active sites of the four target enzymes (SOD, CAT, GR and GPx). Interestingly, the free energy of the binding of the ions (Zn^2+^ and As^3+^) were found to be comparable to each other for all the targets, and quite lower than the metabolites, AsA and GSH (Table 3). These ions lacked any hydrogen bond-forming group, and thereby showed no hydrogen bonding score, which validates the accuracy of the modelling analysis. The results indicate that Zn^2+^ may potentially interfere with the direct interactions of As^3+^ with the target enzymes (Figure 1, Figure 2, Figure 3 and Figure 4). The free energy of the binding of both these ions at the active sites of all the targets was predicted to be the same. This is because of the similar chemical and physical nature of the two ions. Furthermore, regarding GR, in the case of all the receptors, AsA and GSH may potentially interact at the same site as that of As^3+^ and thereby may interfere with the interaction of the ions (Figure 1, Figure 2 and Figure 4). The higher free energies of the binding (docking scores) of the GSH and AsA with all the receptors is attributable to their larger molecular surfaces for forming more interactions, and also to the hydrogen bond-forming groups present in them [50,51]. 

When a ligand binds at the active site of a receptor/enzyme, it may interfere with its activity, which may be regarded as a direct effect of As^3+^, resulting in oxidative stress; however, Zn^2+^ was found to be potent in ameliorating the effect of As^3+^, by excluding it from the active site of the target enzymes. Moreover, AsA and GSH may confer a similar protection and these two metabolites show a much higher free energy of binding with the receptors. It may be noted that the comparable free energy of the binding of As^3+^ and Zn^2+^, and the much higher scores of AsA and GSH may exclude As^3+^ from interacting with and affecting the activities of the four enzymes. Taken together, the computational modeling revealed a novel mode of toxicity of As^3+^ in rice by directly affecting four key antioxidant enzymes, and it also delineates the potential role of Zn^2+^, AsA and GSH in conferring protection; however, the limitation of the present study is that crystal structures of the four target enzymes were not available, and the modeling study was performed based on structures predicted using homology modeling. In the future, an illustration of the stress response (including the interaction effects) would be much clearer for researchers with the help of such an integrative approach (among the “*wet lab*” and “*dry lab*”).

## 5. Conclusions

From the present investigation, it can be concluded that the seed priming technique using Zn^2+^ is a prospective and useful means for regulating the As-induced stress responses in rice. The outcomes of the physiological and biochemical analyses clearly indicated that Zn^2+^ modulates antioxidant metabolism, which results in restraining a high ROS production and ameliorating oxidative stress. Further, Zn^2+^ was also able to limit the As uptake. The datasets obtained from the computational analysis substantiate our experimental findings (in vitro experiments), thus, clearly indicating As stress amelioration by Zn^2+^ supplementation. The results of the current investigation are reasonably significant and unveil an exciting as well as a useful method for evaluating the abiotic stress responses in plants. 

## Figures and Tables

**Table 1:** Consequences of seed priming with Zn^2+^ on rice seedling growth, chlorophyll content and As-accumulation pattern grown under As stress.

Treatments	Elongation (Root)	Elongation (Shoot)	Fresh Weight (Root)	Fresh Weight (Shoot)	Dry Weight (Root)	Dry Weight (Shoot)	Total Chlorophyll	As Content (Root)	As Content (Shoot)
**Main Effects**									
**Variety**									
Gomti	7.58a	11.40a	3.21a	6.96a	0.016a	0.307a	19.5a	32.3a	25.6a
Kalijeera	7.15a	9.95b	1.81b	3.76b	0.018a	0.138b	23.4a	24.1b	11.4b
**Seed priming with As**									
Control	9.01a	14.09a	3.72a	7.01a	0.029a	0.390a	29.3a	-	-
As	5.77d	8.53c	1.69c	4.07c	0.009c	0.120c	15.9b	30.8a	18.2a
As + Zn (0.5 mg L^−1^)	6.93c	9.42c	2.15b	5.02b	0.015b	0.170bc	19.2b	28.8ab	20.4a
As + An (1.0 mg L^−1^)	7.73b	10.65b	2.49b	5.33b	0.015b	0.209b	21.4b	250b	170a
**Interaction Effects**									
Gomti × Control	10.12a	15.58a	4.22a	8.21a	0.034a	0.596a	24.2ab	-	-
Gomti ×As	6.08cd	8.30d	2.58bc	5.98c	0.006d	0.151bc	12.5b	38.3a	23.9a
Gomti × As × Zn (0.5 mg L^−1^)	6.72bcd	9.80cd	2.90b	6.51bc	0.011cd	0.200bc	19.7b	32.8ab	29.8a
Gomti × As × Zn (1.0 mg L^−1^)	7.38bc	11.92bc	3.16b	7.12ab	0.013cd	0.281b	21.5b	26.0bc	23.2a
Kalijeera × Control	7.90b	12.60b	3.22ab	5.80c	0.025ab	0.184bc	34.3a	-	-
Kalijeera × As	5.46d	8.76d	0.80e	2.17e	0.013cd	0.089c	19.3b	23.3c	12.6b
Kalijeera × As × Zn (0.5 mg L^−1^)	7.14bc	9.04d	1.41de	3.54d	0.018bc	0.141c	18.6b	24.9bc	10.9b
Kalijeera × As × Zn (1.0 mg L^−1^)	8.08b	9.38d	1.82cd	3.54d	0.016bcd	0.136c	21.2b	24.0bc	10.8b
**ANOVA**									
**Source of variation**									
**ANOVA *p* Values**									
Variety	0.1010	0.0007	<0.0001	<0.0001	0.2881	<0.0001	0.0524	<0.0001	<0.0001
Seed priming	<0.0001	<0.0001	<0.0001	<0.0001	<0.0001	<0.0001	0.0002	0.0323	0.1754
Variety × seed priming	0.0012	0.0130	0.3651	0.0299	0.0019	<0.0001	0.1312	0.0161	0.0837

# Values are mean (*n* = 5). Values in the column bearing similar letter cases are not significant at the 0.05 level.

**Table 2:** Consequences of seed priming with Zn on seedling oxidative stress-induced biomarkers and enzymatic profile of rice seedling grown under As stress.

Treatment	H_2_O_2_ (Root)	H_2_O_2_ (Shoot)	O_2_^●^^−^ (Root)	O_2_^●^^−^ (Shoot)	MDA (Root)	MDA (Shoot)	CAT (Root)	CAT (Shoot)	GPX (Root)	GPX (Shoot)	SOD (Root)	SOD (Shoot)	GR (Root)	GR (Shoot)
**Main Effects**														
**Variety**														
Gomti	48.9b	44.2b	49.5a	39.8a	65.3b	40.4a	42.3a	51.2a	10.73a	8.73a	108.7a	102.9a	7.36a	7.41b
Kalijeera	80.1a	64.4a	45.5a	42.6a	127.7a	42.1a	45.6a	45.8a	4.43bb	4.39b	71.7b	77.7b	6.41b	12.48a
**Seed priming**														
Control	16.9d	11.5c	11.2c	9.3c	27.5d	13.2d	47.8b	50.2b	7.13b	8.17ab	99.2b	116.4a	9.73a	14.76a
As	107.1a	82.5a	71.7a	64.9a	175.0a	75.1a	19.8c	21.4c	2.34c	2.13c	42.4c	44.0c	2.73c	4.81c
As + Zn (0.5 mg L^−1^)	72.4b	63.1b	55.7b	47.5b	103.5b	41.5b	50.9ab	58.4a	9.72a	7.11b	102.6b	100.3b	7.00b	9.90b
As + An (1.0 mg L^−1^)	61.6c	60.1b	51.4b	43.2b	80.0c	35.1c	57.2a	63.8a	11.13a	8.85a	116.7a	100.5b	8.08b	10.32b
Interaction effects														
Gomti × Control	17.5de	12.4c	9.1d	7.9d	24.1c	14.8c	28.5cd	37.6de	7.13c	9.69b	100.2b	112.7b	9.69a	14.29a
Gomti × As	95.5b	77.1a	86.8a	70.9a	134.1b	70.6a	16.1d	20.5e	2.68d	2.16d	43.7cd	37.2e	1.89d	2.22b
Gomti × As x Zn (0.5 mg L^−1^)	43.7c	48.6b	59.3bc	45.1bc	58.7c	42.4b	53.6ab	64.6ab	14.84b	10.17ab	131.7a	128.1ab	8.12ab	6.14b
Gomti × As × Zn (1.0 mg L^−1^)	38.9cd	38.5b	42.8c	35.5c	44.4c	33.6b	70.8a	81.9a	18.27a	12.91a	159.2a	133.5a	9.74a	6.99b
Kalijeera × Control	16.4e	10.5c	13.3d	10.7d	31.0c	11.6c	67.1a	62.9bc	7.12c	6.64c	98.2b	120.0ab	9.78a	15.23a
Kalijeera × As	118.6a	88.0a	56.7bc	59.0ab	216.0a	79.6a	23.4d	22.4e	2.01d	2.10d	41.1d	50.9de	3.57cd	7.39b
Kalijeera × As × Zn (0.5 mg L^−1^)	101.0ab	77.5a	52.1bc	49.8b	148.3b	40.6b	48.2b	52.2bcd	4.60cd	4.05cd	73.4bc	72.6c	5.88bc	13.67a
Kalijeera × As ×Zn (1.0 mg L^−1^)	84.2b	81.6a	59.9b	51.0b	115.6b	36.6b	43.5bc	45.6cd	3.99cd	4.78cd	74.1b	67.4cd	6.42bc	13.65a
**ANOVA**														
**Source of variation**														
**ANOVA *P* values**														
Variety	<0.0001	<0.0001	0.1254	0.2156	<0.0001	0.3201	0.2499	0.0626	<0.0001	<0.0001	<0.0001	<0.0001	0.0498	<0.0001
Seed priming	<0.0001	<0.0001	<0.0001	<0.0001	<0.0001	<.0001	<0.0001	<0.0001	<0.0001	<0.0001	<0.0001	<0.0001	<0.0001	<0.0001
Variety × seed priming	<0.0001	<0.0001	<0.0001	0.0015	0.0002	0.0719	<0.0001	<0.0001	<0.0001	<0.0001	<0.0001	<0.0001	0.0025	0.0335

# Values are mean (*n* = 5), values bearing the same letter cases are not significantly different at the 0.05 level.

**Table 3:** AsA and GSH content in roots and shoots of rice seedlings.

Treatment	AsA (Root)	AsA (Shoot)	GR/GSH (Root)	GR/GSH (Shoot)
**Main Effects**				
*Variety*				
Gomti	23.5b	33.1a	82.6a	46.1b
Kalijeera	28.3a	33.4a	78.0a	52.7a
*Seed priming*				
Control	36.1a	48.8a	110.0a	90.6a
As	14.4d	18.7d	43.9d	19.5d
As + Zn (0.5 mg L^−1^)	23.2c	29.3c	73.5c	37.0c
As + An (1.0 mg L^−1^)	29.9b	36.2b	93.7b	50.5b
***Interaction Effects***				
Gomti × Control	31.8b	51.2a	111.0a	82.0a
Gomti × As	12.3e	18.3d	32.3c	19.9d
Gomti × As × Zn (0.5 mg L^−1^)	21.2cd	28.4cd	80.7ab	28.4cd
Gomti × As × Zn (1.0 mg L^−1^)	28.9bc	34.4c	106.1a	54.3b
Kalijeera × Control	40.5a	46.4ab	109.0a	99.3a
Kalijeera × As	16.5de	19.1d	55.5bc	19.1d
Kalijeera × As × Zn (0.5 mg L^−1^)	25.3bc	30.2c	66.2b	45.6bc
Kalijeera × As × Zn (1.0 mg L^−1^)	30.9b	37.9bc	81.3ab	46.8bc
**ANOVA**				
**Sources of Variations**				
**ANOVA *p*-value**				
Variety	0.0007	0.8447	0.3557	0.0397
Seed priming	<0.0001	<0.0001	<0.0001	<0.0001
Variety × seed priming	0.3103	0.3570	0.0098	0.0118

# Values are mean (*n* = 5). Values bearing the same letter cases are not significantly different at the 0.05 level.

**Table 4:** Docking scores of the ligands at the active sites of the receptors. The scores were obtained following docking using MVD, and are the predicted free energy of the binding of the ligands with the receptor, in kcal/mol.

Receptor	Ligands	MolDock Score	Rerank Score	HBond
**Superoxide dismutase (Mn-SOD)**	Superoxide radical	−18.6347	−16.1787	−3.10127
	As^3+^	−19.8086	−20.6329	0
	Zn^2+^	−19.8081	−20.6325	0
	AsA	−80.6404	−71.5325	−9.56283
	GSH	−114.497	−98.2422	−10.4638
**Catalase (CAT)**	H_2_O_2_	−18.8881	−16.2571	−5.68477
	As^3+^	−21.7768	−21.3154	0
	Zn^2+^	−20.6794	−20.1665	0
	AsA	−65.3445	−61.1279	−10.3087
	GSH	−85.3666	−73.7135	−7.49339
**Glutathione reductase (GR)**	As	−27.7162	−19.5761	0
	Zn	−28.2799	−24.9889	0
	AsA	−79.9338	−71.3906	−9.22549
	GSH	−116.038	−100.133	−11.5623
**Glutathione peroxidase (GPx)**	H_2_O_2_	−20.0855	−17.0414	−5.25808
	As^3+^	−22.9651	−24.3977	0
	Zn^2+^	−22.9649	−24.3974	0
	AsA	−74.8541	−68.4545	−9.85995
	GSH	−106.223	−92.9154	−14.2971

## Data Availability

The data that support the findings of this study are openly available on request.
